# Patterns of germline and somatic testing after universal tumor screening for Lynch syndrome: A clinical practice survey of active members of the Collaborative Group of the Americas on Inherited Gastrointestinal Cancer

**DOI:** 10.1002/jgc4.1567

**Published:** 2022-02-26

**Authors:** Rachel Hodan, Linda Rodgers‐Fouche, Sanjeevani Arora, Mev Dominguez‐Valentin, Priyanka Kanth, Bryson W. Katona, Kathryn A. Mraz, Maegan E. Roberts, Eduardo Vilar, Cynthia M. Soto‐Azghani, Randall E Brand, Edward D. Esplin, Kimberly Perez

**Affiliations:** ^1^ Cancer Genetics Stanford Health Care Palo Alto California USA; ^2^ Center for Cancer Risk Assessment Massachusetts General Hospital Boston Massachusetts USA; ^3^ Cancer Prevention and Control Program Fox Chase Cancer Center Philadelphia Pennsylvania USA; ^4^ Department of Tumor Biology Institute of Cancer Research The Norwegian Radium Hospital Oslo Norway; ^5^ Division of Gastroenterology University of Utah Health and Huntsman Cancer Institute Salt Lake City Utah USA; ^6^ Division of Gastroenterology and Hepatology University of Pennsylvania Perelman School of Medicine Philadelphia Pennsylvania USA; ^7^ Center for Genomic Interpretation Sandy Utah USA; ^8^ Grey Genetics, LLC Brooklyn New York USA; ^9^ GeneDx Gaithersburg Maryland USA; ^10^ Department of Clinical Cancer Prevention The University of Texas MD Anderson Cancer Center Houston Texas USA; ^11^ Independent ‐ Clinical Research Nurse Tyler Texas USA; ^12^ Gastrointestinal Malignancy Early Detection, Diagnosis and Prevention Program University of Pittsburgh Pittsburgh Pennsylvania USA; ^13^ Invitae San Francisco California USA; ^14^ Department of Medical Oncology Dana‐Farber Cancer Institute/ Harvard Medical School Boston Massachusetts USA

**Keywords:** clinical practice barriers, genetic counseling, genetic testing, Lynch syndrome, risk assessment, universal tumor screening

## Abstract

Clinical guidelines recommend universal tumor screening (UTS) of colorectal and endometrial cancers for Lynch syndrome (LS). There are limited guidelines for how to integrate germline testing and somatic tumor testing after a mismatch repair deficient (dMMR) tumor is identified. We sought to characterize current practice patterns and barriers to preferred practice among clinical providers in high‐risk cancer programs. A clinical practice survey was sent to 423 active members of the Collaborative Group of the Americas on Inherited Gastrointestinal Cancer (CGA‐IGC) with a follow‐up survey sent to 103 clinician responders. The survey outlined clinical vignettes and asked respondents their preferred next test. The survey intended to assess: (1) the role of patient age and family history in risk assessment and (2) barriers to preferred genetic testing. Genetic test options included targeted germline testing based on dMMR expression, germline testing for LS, germline testing with a multigene cancer panel including LS, or paired tumor/germline testing including LS. In October 2020, 117 of 423 (28%) members completed the initial survey including 103 (88%) currently active clinicians. In April 2021, a follow‐up survey was sent to active clinicians, with 45 (44%) completing this second survey. After selecting their preferred next germline or paired tumor/germline tumor test based on the clinical vignette, 39% of respondents reported wanting to make a different choice for the initial genetic test without any testing barriers. The proportion of respondents choosing a different initial genetic test was dependent on the proband's age at diagnosis and specified family history. The reported barriers included patient's lack of insurance coverage, patient unable/unwilling to self‐pay for proposed testing, and inadequate tumor tissue. Responders reported insurance, financial constraints, and limited tumor tissue as influencing preferred genetic testing in high‐risk clinics, thus resulting in possible under‐diagnosis of LS and impacting potential surveillance and cascade testing of at‐risk relatives.

**What is known about this topic**

A survey of practicing clinicians about subsequent testing after universal tumor screening in the era of both germline and somatic clinically available testing has not been studied to our knowledge.

**What this paper adds to the topic**

A general understanding of practice patterns among high‐volume clinicians mostly in the United States, given the lack of clinical guidelines in this area. This study also addresses perceived barriers to preferred genetic testing in the setting of universal tumor screening.

## 1 BACKGROUND/INTRODUCTION

Clinical guidelines recommend universal tumor screening (UTS) of colorectal (CRC) and endometrial cancers (EC) to improve the identification of individuals and families with Lynch syndrome (LS) (NCCN, 2021). UTS can be accomplished by assessing the expression of the mismatch repair (MMR) proteins (MLH1, MSH2, MSH6, PMS2) by immunohistochemistry (IHC) and/or by PCR‐based microsatellite instability (MSI) analysis in tumor tissue. LS (formerly known as hereditary nonpolyposis colorectal cancer; HNPCC) is caused by germline pathogenic variants in *MLH1, MSH2, MSH6, PMS2* or by deletion of the 3’ end of *EPCAM* (*TACSTD1*), resulting in hypermethylation of the *MSH2* promoter (Ligtenberg et al., 2009), each of which results in different risks for cancer, particularly CRC, EC, and ovarian cancer. The absence of at least one protein is indicative for underlying MMR deficiency (dMMR) and can represent one of the following etiologies: (1) sporadic *MLH1* promoter methylation; (2) a germline pathogenic variant in LS‐associated genes plus a second somatic ‘hit’; (3) biallelic somatic mutations; (4) error in the original screen; or (5) unexplained dMMR. Aside from these potential outcomes, false‐negative results also limit UTS with sensitivity of IHC reported at 83% for subsequently identifying *MLH1*, *MSH2*, or *MSH6* pathogenic variants, and sensitivity of MSI at 87% for subsequently identifying pathogenic *MLH1* or *MSH2* variants and 77% for *MSH6* (Palomaki et al., 2009 *)*. A wide yield of 12%–67% of patients with dMMR tumors not explained by *MLH1*‐hypermethylation are found to have a germline pathogenic variant in a MMR gene, with the yield for CRC 11%–58% (Adar et al., 2018; Pearlman et al., 2019) and for EC 29%–45% (Adar, 2018; Ryan et al., 2019).

The National Comprehensive Cancer Network (NCCN) recommends UTS of all CRC and EC. When dMMR not explained by *MLH1*‐hypermethylation is identified, NCCN recommends initial germline testing or paired tumor/germline and then the consideration, but not recommendation, for somatic MMR testing if germline testing is negative (NCCN guidelines, 2021). NCCN does not make a definitive recommendation to incorporate somatic tumor testing (either separately or as paired tumor/germline analysis) for the purpose of identifying biallelic somatic mutations. Given the limitations of UTS, barriers to genetic assessment, and lack of consensus on how to integrate UTS results with somatic and germline testing for LS, we sought to characterize current practice patterns among clinical providers in high‐risk cancer programs by surveying active members of The Collaborative Group of the Americas on Inherited Gastrointestinal Cancer 2021 (CGA‐IGC).

## 2 METHODS

### 2.1 Population

CGA‐IGC is an organization with the mission to advance science and clinical care of inherited gastrointestinal cancers through research and education in the Americas (Collabortive Group of the Americas on Inherited Gastrointestinal Cancer mission statement, 2021). In 2020–2021, there were 423 active members from 29 states in the US and 11 countries. Membership includes physicians, scientists, allied health professionals, genetic counselors, and trainees.

### 2.2 Survey structure

This study was reviewed by the Institutional Review Board at Stanford University and given exempt status. A clinical practice survey was initially submitted to 423 CGA‐IGC active members, using SurveyMonkey (https://www.surveymonkey.com). The survey included assessment of demographics, clinical practice specialty, established institutional LS diagnostic practices, and eight clinical vignettes intended to assess: (a) the role of patient age at diagnosis of cancer and family history on genetic risk assessment; (b) role of patient age at diagnosis of cancer, family history, somatic tumor and/or germline test results on CRC and gynecologic surveillance.

A follow‐up survey was sent to the initial group of 103 responders who identified themselves as active clinicians to assess practice barriers based on the initial four vignettes that included questions specific to test ordering patterns. The survey included the identical initial four clinical vignettes with follow‐up questions intended to assess: (a) the role of patient age and family history in genetic risk assessment and testing; and (b) barriers to genetic assessment. Testing options included targeted germline testing based on dMMR expression results, germline testing for the five genes associated with LS, germline testing with a multigene cancer panel including LS gene, or paired tumor/germline testing including LS genes.

The following background information and case details were given to the respondents. The initial survey is included in File S1 and the follow‐up survey is included in File S2.

For all vignettes, tumor screening was performed by MMR immunohistochemistry (IHC) for MLH1, MSH2, MSH6, and PMS2 proteins. The CRC of the proband had paired loss of MSH2/MSH6. Paired tumor/germline testing includes both tumor sequencing and germline testing of at least the five Lynch syndrome (LS) genes (*MLH1, MSH2, EPCAM, MSH6, PMS2*). It may also include somatic or germline testing of additional non‐LS genes.

For each vignette, respondents were asked to indicate their typical next test ordered. The vignettes were purposefully similar to one another to elucidate whether the combination of age of diagnosis of the CRC in the proband or family history of CRC at age 50 changed the test ordering patterns. Respondents could choose more than one barrier if they responded ‘yes’ to the question of a change in test ordering because of barriers.

## 3 RESULTS

### 3.1 Survey response

Nearly one‐third (117/423; 28%) of CGA‐IGC active members completed the initial survey in October 2020. The majority (103/117; 88%) of respondents confirmed they were practicing clinicians treating patients with inherited gastrointestinal cancer. Close to half (45/103; 44%) of the original clinician respondents completed a second survey in April 2021. Results below are presented for the second group of respondents (*N* = 45).

#### 3.1.1 Demographics, clinical practice specialty and established institutional LS diagnostic practice characteristics

Respondents for the follow‐up survey identified themselves as clinicians in academic medical centers (60%), non‐academic medical centers (36%), in private practice (2%), or other practice type (2%). Respondents were genetic counselors in cancer genetics (62%), genetic counselors specifically practicing in only gastrointestinal cancer genetics (11%), oncologists (7%), gastroenterologists (13%), geneticists (4%) or colorectal surgeons (2%). Most (37/45; 82%) respondents reported evaluating at least 10 patients a month for hereditary gastroenterology risk assessment. The survey did not ask respondents the volume of germline or paired tumor/germline tests being ordered. The majority of respondents practice in the United States (40/45; 89%). Demographics are similar for the initial survey clinician respondents (*N* = 103) and the follow‐up survey clinician respondents (*N* = 45). Complete demographics are outlined in Table 1.

**TABLE 1:** Demographics of clinician respondents for initial and follow up survey

Respondents	Clinician respondents from initial survey (*N* = 103) *N* (%)	Clinician respondents from follow up survey (*N* = 45) *N* (%)
Specialty		
Genetic counselor in cancer genetics	65 (63)	28 (62)
Genetic counselor in hereditary gastroentestinal cancer only	8 (8)	5 (11)
Gastroentestinal oncologist	3 (3)	1 (2)
Medical oncologist	2 (2)	2 (4)
Gastroenterologist	17 (16)	6 (13)
Medical geneticist	4 (4)	2 (4)
Colorectal surgeon	2 (2)	1 (2)
Other	2 (2)	0 (0)
Practice location		
USA	89 (86)	40 (89)
Canada	9 (9)	3 (7)
Italy	2 (2)	1 (2)
Other	3 (3)	1 (2)
Practice type		
Academic medical center	75 (73)	27 (60)
Non‐academic medical center	19 (18)	16 (36)
Private practice	7 (7)	1 (2)
Other	2 (2)	1 (2)
Number of patients seen per month for hereditary cancer risk assessment		
1–5	21 (20)	7 (16)
6–10	40 (39)	15 (33)
11–20	24 (23)	16 (36)
21–30	10 (10)	3 (7)
31–40	5 (5)	2 (4)
41–50	1 (1)	1 (2)
No response	2 (2)	1 (2)

### 3.2 Barriers for preferred LS genetic assessment

Most clinicians (42/45; 93%) responded to the first clinical vignette. About half of the respondents (20/42; 48%) stated their typical next test would be paired tumor/germline with the exact same number (20/42; 48%) stating they would start with germline testing only, with two respondents reporting an ‘other’ testing choice (2/42; 5%). Germline testing was considered in aggregate and included targeted *MSH2/MSH6/EPCAM* testing, five gene LS testing, or germline testing with a multigene panel which included the five genes associated with LS. Thirty‐nine percent (16/41) responded that their answer would have been different without barriers. The most commonly identified barriers included lack of insurance coverage (10/16; 63%), patient unwilling to self‐pay if insurance does not cover (11/16; 69%), and/or tumor unavailable for paired analysis (9/16; 56%). A small number of respondents indicated a barrier of turnaround time for paired analysis (4/16; 25%) and non‐flexibility to decide their ordering pattern of testing (3/16; 19%). No respondents indicated a discomfort in counseling about somatic test results.

Thirty‐nine of the 45 (87%) clinicians responded to the second clinical vignette. Again, about half of the respondents (20/39; 51%) stated their typical next test would be paired tumor/germline and half (19/39; 49%) stated they would start with germline testing only. With the addition of family history, only ten respondents (10/39; 26%) replied that their answer would have been different without barriers. The most commonly identified barriers were again lack of insurance coverage (9/10; 90%), patient unwilling to self‐pay if insurance does not cover (9/10; 90%), and/or tumor unavailable for paired analysis (6/10; 60%).

Eighty‐four percent (38/45) of clinicians responded to the third vignette. A similar initial pattern of test ordering was identified with 50% (19/38) and 47% (18/39) of respondents choosing paired tumor/germline and germline only testing, respectively. Approximately one‐third (13/38; 34%) of respondents indicated they would have made a different choice for the initial test without barriers. This is similar to the first vignette which also featured a proband without family history. Similar barriers were reported including lack of insurance coverage (10/13; 77%), patients unwilling to self‐pay if insurance does not cover (9/13; 69%), and/or tumors unavailable for paired analysis (7/13; 54%). Eighty‐four percent (38/45) of clinicians responded to the fourth vignette. Sixteen respondents (16/38; 42%) chose upfront paired tumor/germline and only nine respondents indicated they wanted to order a different initial test (9/38; 24%) with the addition of family history.

### 3.3 The role of patient age and family history in genetic risk assessment and initial genetic testing

Respondents noted a higher desire to change their initial test if there was no reported family history, independent of age of proband with a dMMR CRC; 39% (16/41) for the 45‐year‐old proband and 34% (13/38) for the 75‐year‐old proband. Barriers were less impactful in the scenarios in which there was a reported cancer family history, with 26% (10/39) and 24% (9/38) of respondents wanting to change their initial test for younger proband and older proband, respectively (summarized in Table 2).

**TABLE 2:** Vignette characteristics and responses

Vignette	Age of proband with dMMR CRC	Family history	Respondents *N* (%)	Initial test germline only *N* (%)	Initial test paired tumor/germline *N* (%)	Respondents who said their initial test would have been different without barriers *N* (%)
1	45	None	42 (93)	20 (48)	20 (48)	16 (39)
2	45	Mother with CRC at 50	39 (87)	19 (49)	20 (51)	10 (26)
3	75	None	38 (84)	18 (47)	19 (50)	12 (34)
4	75	Mother with CRC at 50	38 (84)	21 (55)	16 (42)	9 (24)

Note

Germline testing included options for targeted *MSH2/MSH6/EPCAM* testing, LS‐associated gene testing or a multigene panel. Paired tumor/germline testing included both somatic and germline analysis of pathogenic variants in the LS genes.

Abbreviations: CRC, colorectal cancer; dMMR, mismatch repair deficient.

## 4 DISCUSSION

This was an inaugural exploratory study conducted by the Research Committee of the CGA‐IGC to understand practice patterns of its clinician members. As an international organization with a mission to advance science and clinical care of inherited gastrointestinal cancers through research and education, the CGA‐IGC clinician members’ practice patterns were of high interest. Most respondents practice in the United States and perform hereditary risk assessment for a high volume of patients, allowing some potential generalizability of the data for United States high‐volume clinicians. More than 70% of the respondents were genetic counselors when combining genetic counselors in cancer genetics and those specifically practicing in gastrointestinal cancer genetics, highlighting that germline and somatic tumor testing ordering patterns may be relevant for cancer genetic counselors.

There is uncertainty in how and when to order germline and/or somatic tumor testing after UTS, even for clinicians with expertise in hereditary cancer risk assessment. This uncertainty is highlighted by the classic scenario of a patient with CRC with loss of MSH2/MSH6 on MMR IHC staining and no germline pathogenic variant in an MMR gene. Patients and their family members with this MMR staining pattern were previously recommended to follow LS management, even without the identification of a germline pathogenic variant in an MMR gene (Weissman et al., 2012). This scenario can be clarified in some cases with tumor testing and the identification of biallelic somatic mutations as an etiology of tumors with dMMR staining (Geurts‐Giele et al., 2014; Haraldsdottir et al., 2014; Mensenkamp et al., 2014). To account for this potential scenario, the diagnostic process has been streamlined by the commercial availability of paired tumor/germline testing in the setting of dMMR after UTS, however, barriers exist to utilize this as first‐line testing after UTS.

In our survey cohort, respondents were evenly split on preference of initial assessment with paired tumor/germline versus germline alone when including all germline testing options in aggregate. The notable difference in practice patterns was demonstrated when a comparison was made assessing test preferences with the presence or absence of family history. Respondents noted a higher desire to change their initial response if there was no reported family history independent of age of proband with a dMMR CRC, presumably to obtain additional somatic tumor information. Similarly, barriers were less impactful in the scenarios in which there was a reported family history since more clinicians chose upfront germline only. This difference is not unexpected, since in the setting of both a proband with a dMMR CRC and family history, the etiology is more likely to be germline (Pearlman et al., 2019) and thus somatic tumor testing is presumably of less importance.

These results suggest that clinicians practicing in high‐risk programs want to utilize paired tumor/germline testing more than they are currently doing in the setting of dMMR after UTS. This would be presumably for the purposes of better clarification of dMMR etiology to guide medical management recommendations for the proband and their family. Recently, Kaiser Permanente (Carwana et al., 2021) analyzed the efficacy of using paired germline/tumor analysis in a cohort of patients meeting criteria of Lynch‐like syndrome (defined as dMMR with no germline MMR pathogenic variant). Paired tumor/germline testing further characterized 68% of the cohort, identifying 31 (62%) patients with sporadic cancer and 3 (6%) patients with LS. These results, as well as the interest demonstrated by our cohort, support future studies to identify the optimal testing algorithm for LS assessment.

Multiple studies have suggested utilization of UTS is more cost efficient when compared to targeted germline testing (Di Marco et al., 2018). The most commonly reported barriers in all clinical vignettes were lack of insurance coverage, patient unwilling to self‐pay if insurance does not cover, or tumor unavailable for paired analysis. Taken in aggregate, the barrier of coverage or cost is by far the most commonly reported issue. These results highlight the concern regarding cost efficiency of paired testing compared to germline testing alone, which has not yet been assessed. Future studies are needed to determine if paired testing improves cost efficiency relative to germline testing alone, if it improves the number of relatives detected through cascade screening, and how much decision‐makers are willing to pay for modest improvements in case detection. The combination of efficacy and cost‐effectiveness may guide a different incorporation of paired tumor/germline testing in algorithms for clinical guidelines. Additional barriers to be studied that are not addressed here include access to genetic counseling and/or genetic testing for hereditary risk assessment.

The main limitation of our study is that the majority of our respondents are clinicians in high‐volume high‐risk programs, so the barriers indicated may be specific to this population. Future work could survey clinician members of larger societies.

In summary, our data demonstrate perceived barriers to accessing optimal genetic testing to clarify the etiology of dMMR CRC, especially in the setting of a proband with dMMR and no family history. Understanding the current practice patterns of high‐volume clinicians may assist clinical guidelines committees or organizational practice guidelines to incorporate more specific recommendations for testing after UTS. This would allow for better risk assessment for both probands and at‐risk relatives and allow for subsequent opportunities for cancer screening and prevention in LS.

## AUTHOR CONTRIBUTIONS

Authors Hodan and Perez confirm that they had full access to all the data in the study and take responsibility for the integrity of the data and the accuracy of the data analysis. All authors gave final approval of this version to be published and agree to be accountable for all aspects of the work in ensuring that questions related to the accuracy or integrity of any part of the work are appropriately investigated and resolved.

Rachel Hodan and Linda Rodgers‐Fouche contributed to conceptualization, data curation, formal analysis, methodology, writing—original draft, and writing—review and editing; Sanjeevani Arora, Priyanka Kanth, Bryson W. Katona, Kathryn A. Mraz, Maegan E. Roberts, Cynthia M. Soto‐Azghani, and Edward D. Esplin contributed to conceptualization, methodology, and writing—review and editing; Mev Dominguez‐Valentin contributed to conceptualization, methodology, writing—original draft, and writing—review and editing; Eduardo Vilar contributed to conceptualization and writing—review and editing; Randall E Brand contributed to conceptualization, methodology, supervision, and writing—review and editing; Kimberly Perez contributed to conceptualization, formal analysis, methodology, writing—original draft, and writing—review and editing.

## COMPLIANCE WITH ETHICAL STANDARDS

### Conflict of Interest

Hodan, Rodger‐Fouche, Dominguez‐Valentin, Kanth, Katona, Mraz, Vilar, Soto‐Azghani, and Brand confirm that they have no conflicts of interest.

### Human studies and informed consent

This study was reviewed and granted an exemption by the Stanford institutional review board. All procedures followed were in accordance with the ethical standards of the responsible committee on human experimentation (institutional and national) and with the Helsinki Declaration of 1975, as revised in 2000. Implied informed consent was obtained for individuals who voluntarily completed the online survey and submitted their responses.

### ANIMAL STUDIES

No non‐human animal studies were carried out by the authors for this article.

### DATA AVAILABILITY STATEMENT

The data that support the findings of this study are available from the corresponding author upon reasonable request.

## Supporting information

File S1

File S2
