# Supplementary material for: Patterns of germline and somatic testing after universal tumor screening for Lynch syndrome: A clinical practice survey of active members of the Collaborative Group of the Americas on Inherited Gastrointestinal Cancer
Source: J Genet Couns. 2022 Feb 26;31(4):949–55. doi: 10.1002/jgc4.1567 (PMC9544955; doi:10.1002/jgc4.1567)
Supplement: Supplementary file 1 — File S1 [file JGC4-31-949-s001.docx]

CGA Clinical Practice Survey 2020

Demographics

1. What is your primary work setting?
   1. Academic Medical Center
   2. Non-academic medical center
   3. Private practice
   4. Other (fill in)
2. What is your primary specialty/role? [DROP DOWN MENU]
   1. Genetic Counselor in Cancer Genetics
   2. Genetic Counselor in Hereditary GI only
   3. Gastroenterologist
   4. GI Oncologist
   5. Medical oncologist
   6. Colorectal surgeon
   7. Thoracic surgeon
   8. Surgical oncologist
   9. General surgeon
   10. Gynecologic oncologist
   11. Gynecologist
   12. Primary care provider
   13. Researcher
   14. Nurse
   15. Medical Geneticist
   16. Other (fill in)
3. Location of primary practice

[Drop-down menu of all countries]

[If USA chosen, drop down menu for each state]

1. Approximate average number of patients seen *per month* by you for initial (i.e. new patient) hereditary gastrointestinal (GI) cancer risk assessment. *Note: this question is asking your individual volume and not the practice/clinic volume.*
   1. 0-*I do not see patients for hereditary GI cancer risk assessment* [If this is chosen, have the survey skip question 5]
   2. 1-5
   3. 6-10
   4. 11-20
   5. 21-30
   6. 31-40
   7. 41-50
   8. 51-60
   9. 60+
2. What year was your practice/group established for hereditary GI cancer risk assessment?

[Drop down menu with years from 1980-present]

Current Lynch Syndrome (LS) screening and testing practices

For the purposes of this survey, universal tumor screening for Lynch Syndrome (LS) is defined as any *automatic* isolated or combined tumor screening approach including microsatellite instability (MSI) or mismatch repair (MMR) immunohistochemistry (IHC) with or without secondary reflex testing with BRAF and/or MLH1 promotor hypermethylation testing, that is performed on at least one type of LS-related cancer (colorectal, endometrial, etc.).

1. Does your hospital/practice participate in *automatic* universal tumor screening for LS?
   1. Yes
   2. No

[If Yes, continue to answer the following]

1. What year was *any type* of universal screening for LS established?
   1. Drop down menu with list of years
2. *Regarding your current practice,* select the tumor types and applicable ages for universal screening:

| Tumor type | Not included in universal screening | All tumors, regardless of age | All tumors diagnosed at age 50 or younger screened | All tumors diagnosed at age 70 or younger | Other (write-in details) |
| --- | --- | --- | --- | --- | --- |
| Colorectal |  |  |  |  |  |
| Uterine/endometrial |  |  |  |  |  |
| Ovarian |  |  |  |  |  |
| Gastric |  |  |  |  |  |
| Pancreatic |  |  |  |  |  |
| Small bowel |  |  |  |  |  |
| Renal/renal pelvic |  |  |  |  |  |
| Other urinary tract |  |  |  |  |  |
| Hepatobiliary tract |  |  |  |  |  |
| Sebaceous neoplasms |  |  |  |  |  |
| Other (write-in) |  |  |  |  |  |

1. What type of universal tumor screening is the *initial test:*
2. Immunohistochemistry (IHC) of MLH1, MSH2, MSH6 and PMS2
3. Immunohistochemistry (IHC) of MSH6 and PMS2 only
4. PCR-based microsatellite instability (MSI) testing
5. Other (fill in)
6. If IHC for all four proteins is the *initial* test and a colorectal tumor shows MLH1/PMS2 loss on staining, which of the following is an *automatic* reflex test
   1. BRAF v600E analysis
   2. MLH1 promotor hypermethylation
   3. Both BRAF v600E analysis *and* MLH1 promotor hypermethylation concurrently
   4. PCR-based MSI testing
   5. No reflex testing performed; clinician’s discretion to order additional testing
   6. No reflex testing performed; clinician’s discretion to refer for germline risk assessment
   7. No reflex testing performed; automatic referral for germline risk assessment
   8. N/A (IHC for all four proteins is not the initial test performed at my hospital/practice)
   9. Other (fill in)
7. If IHC for all four proteins is the *initial* test and an endometrial tumor shows MLH1/PMS2 loss on staining, which of the following is an *automatic* reflex test
   1. BRAF v600E analysis
   2. MLH1 promotor hypermethylation
   3. Both BRAF v600E analysis *and* MLH1 promotor hypermethylation concurrently
   4. PCR-based MSI testing
   5. No reflex testing performed; clinician’s discretion to order additional testing
   6. No reflex testing performed; clinician’s discretion to refer for germline risk assessment
   7. No reflex testing performed; automatic referral for germline risk assessment
   8. N/A (IHC for all four proteins is not the initial test performed at my hospital/practice)
   9. Other (fill in)

Please answer the following questions regarding *automatic* (not clinician initiated after risk assessment) initial or subsequent germline and/or somatic tumor testing. LS germline testing includes analysis by sequencing and deletion/duplication analysis of *MLH1, MSH2, MSH6, PMS2* and *EPCAM* (often del/dup only). Paired tumor/germline testing includes both tumor sequencing and germline testing of at least the LS genes. It may also include somatic or germline testing of additional non-LS genes.

1. Does your hospital/practice perform *automatic* germline or somatic tumor testing for LS?
   1. Yes
   2. No

If yes, please answer the following questions.

1. What type of germline or somatic tumor test is *automatic* and in what context? This could include automatic testing for one cancer type (e.g. colon), but not another (e.g. endometrial)
   1. Tumor sequencing that includes MSI testing performed for all tumors as the initial test (i.e. IHC/MSI not performed separately).
   2. Tumor sequencing that does not include MSI testing performed for all tumors as the initial test (i.e. IHC/MSI not performed separately)
   3. Tumor sequencing performed for all tumors regardless of IHC/MSI results (either concurrently or as reflex to IHC/MSI testing)
   4. Tumor sequencing performed for all tumors with suggestive IHC or MSI results on initial screen (i.e. no automatic germline testing included).
   5. Paired tumor/germline testing performed for all LS-associated tumors as the initial test (i.e. IHC/MSI not performed separately).
   6. Paired tumor/germline testing performed for all tumors with suggestive IHC or MSI results on initial screen
   7. Germline testing performed regardless of IHC/MSI results
   8. Germline testing performed after suggestive IHC or MSI results on initial tumor screen
   9. Other (write-in)
2. Please write any details about your somatic/germline testing process. For example, our hospital performs automatic germline testing only for ovarian cancers, but not for endometrial and colorectal. [Write-in, optional question]
3. If tumor sequencing only (no germline testing) is an *automatic* test, which scenarios would typically lead to a genetics referral for consideration of germline testing for a patient with colorectal cancer:
4. A pathogenic variant/likely pathogenic variant (PV/LPV) in a LS gene on tumor sequencing regardless of microsatellite testing
5. A PV/LPV in a LS gene AND the tumor is MSI-High or has absent MMR
6. A VUS in a LS gene regardless of MSI or MMR status
7. A VUS in a LS gene AND the tumor is MSI-H or has absent MMR
8. Other (fill-in)
9. N/A

For the following case examples, initial screening was performed by IHC for MLH1, MSH2, MSH6 and PMS2 proteins and there are no barriers to testing (referral to genetics, coverage, compliance, insurance, etc.). Paired tumor/germline testing includes both tumor sequencing and germline testing of at least the LS genes. It may also include somatic or germline testing of additional non-LS genes.

1. Your patient was diagnosed with colorectal cancer at age 45 and the tumor showed absent MSH2/MSH6. There is no family history of LS cancers. The typical next test ordered for your patient is:
2. Germline testing for MSH2 only
3. Germline testing for MSH2/MSH6/EPCAM
4. Germline testing for all five LS genes
5. Germline testing with a multi-gene panel that includes all five LS genes
6. Paired tumor/germline testing that includes LS
7. Other (write in)
8. N/A to my practice
9. The above patient had germline testing with a multi-gene panel after the initial IHC screen and tested negative (no pathogenic or likely pathogenic variants identified). The typical next test ordered for your patient is:
   1. No additional testing offered
   2. Tumor sequencing to identify biallelic somatic mutations
   3. PCR-based MSI testing
   4. Other (write-in)
   5. N/A to my practice
10. Your patient was diagnosed with colorectal cancer at age 45 and the tumor showed absent MSH2/MSH6. Your patient’s mother was diagnosed with colorectal cancer at 50 with no additional family history. The typical next test ordered for your patient is:
11. Germline testing for MSH2 only
12. Germline testing for MSH2/MSH6/EPCAM
13. Germline testing for all five LS genes
14. Germline testing with a multi-gene panel that includes all five LS genes
15. Paired tumor/germline testing that includes LS
16. Other (write in)
17. N/A to my practice
18. The above patient had germline testing with a multi-gene panel after the initial IHC screen and tested negative (no pathogenic or likely pathogenic variants identified). The typical next test ordered for your patient is:
    1. No additional testing offered
    2. Tumor sequencing to identify biallelic somatic mutations
    3. PCR-based MSI testing
    4. Other (write-in)
    5. N/A to my practice
19. Your patient was diagnosed with colorectal cancer at age 75 and the tumor showed absent MSH2/MSH6. There is no family history of Lynch cancers. The typical next test ordered for your patient is:
20. Germline testing for MSH2 only
21. Germline testing for MSH2/MSH6/EPCAM
22. Germline testing for all five LS genes
23. Germline testing with a multi-gene panel that includes all five LS genes
24. Paired tumor/germline testing
25. Other (write in)
26. N/A to my practice
27. The above patient had germline testing with a multi-gene panel after the initial IHC screen and tested negative (no pathogenic or likely pathogenic variants identified). The typical next test ordered for your patient is:
    1. No additional testing offered
    2. Tumor sequencing to identify biallelic somatic mutations
    3. PCR-based MSI testing
    4. Other (write-in)
    5. N/A to my practice
28. Your patient was diagnosed with colorectal cancer at age 75 and the tumor showed absent MSH2/MSH6. The patient’s mother had colorectal cancer at age 50. The typical next test ordered for your patient is:
29. Germline testing for MSH2 only
30. Germline testing for MSH2/MSH6/EPCAM
31. Germline testing for all five LS genes
32. Germline testing with a multi-gene panel that includes all five LS genes
33. Paired tumor/germline testing
34. Other (write in)
35. N/A to my practice
36. The above patient had germline testing with a multi-gene panel after the initial IHC screen and tested negative (no pathogenic or likely pathogenic variants identified). The typical next test ordered for your patient is:
    1. No additional testing offered
    2. Tumor sequencing to identify biallelic somatic mutations
    3. PCR-based MSI testing
    4. Other (write-in)
    5. N/A to my practice
37. Your female patient was diagnosed with colorectal cancer at age 50. The tumor showed absent MSH2/MSH6 and subsequent germline testing was negative. There is no family history of LS cancers. Tumor sequencing is not an option. In terms of gynecologic care for this patient:
38. I would not offer any increased surveillance for gynecological cancers
39. I would offer LS gynecologic management options, such as endometrial sampling, transvaginal u/s, but I would not offer TAH/BSO
40. I would offer MSH2-associated LS gynecologic management options, including TAH/BSO
41. Other (fill in)
42. Your female patient was diagnosed with colorectal cancer at age 50. The tumor showed absent MSH2/MSH6 and subsequent germline testing was negative. The family meets Amsterdam I criteria with family history of colorectal cancer only. Tumor sequencing is not an option. In terms of gynecologic care for this patient:
43. I would not offer any increased surveillance for gynecological cancers
44. I would offer LS gynecologic management options, such as endometrial sampling, transvaginal u/s, but I would not offer TAH/BSO
45. I would offer MSH2-associated LS gynecologic management options, including TAH/BSO
46. Other (fill in)
47. Your female patient was diagnosed with colorectal cancer at age 50. The tumor showed absent MSH2/MSH6 and paired tumor/germline testing identified a single somatic *MSH2* PV/LPV. There is no family history of Lynch cancers. In terms of gynecologic care for this patient:
48. I would not offer any increased surveillance for gynecological cancers
49. I would offer LS gynecologic management options, such as endometrial sampling, transvaginal u/s, but I would not offer TAH/BSO
50. I would offer MSH2-associated LS gynecologic management options, including TAH/BSO
51. Other (fill in)
52. Your female patient was diagnosed with colon cancer at age 50. The tumor showed absent MSH2/MSH6 and paired tumor/germline testing identified a single somatic *MSH2* PV/LPV. The family meets Amsterdam I criteria with family history of colorectal cancer only. In terms of gynecologic care for this patient:
53. I would not offer any increased surveillance for gynecological cancers
54. I would offer LS gynecologic management options, such as endometrial sampling, transvaginal u/s, but I would not offer TAH/BSO
55. I would offer MSH2-associated LS gynecologic management options, including TAH/BSO
56. Other (fill in)
