# Supplementary material for: Patterns of germline and somatic testing after universal tumor screening for Lynch syndrome: A clinical practice survey of active members of the Collaborative Group of the Americas on Inherited Gastrointestinal Cancer
Source: J Genet Couns. 2022 Feb 26;31(4):949–55. doi: 10.1002/jgc4.1567 (PMC9544955; doi:10.1002/jgc4.1567)
Supplement: Supplementary file 2 — File S2 [file JGC4-31-949-s002.docx]

CGA Clinical Practice Survey 2020 Part II

[To be sent to all responders from initial study who identified themselves as seeing patients in clinic]

Thank you for participating in the 2020 CGA Clinical Practice Survey. We have some follow up questions for those who participated initially, and we appreciate your willingness to participate again. This survey should take no more than 10 minutes.

Demographics

1. What is your primary work setting?
   1. Academic Medical Center
   2. Non-academic medical center
   3. Private practice
   4. Other (fill in)
2. What is your primary specialty/role? [DROP DOWN MENU]
   1. Genetic Counselor in Cancer Genetics
   2. Genetic Counselor in Hereditary GI only
   3. Gastroenterologist
   4. GI Oncologist
   5. Medical oncologist
   6. Colorectal surgeon
   7. Thoracic surgeon
   8. Surgical oncologist
   9. General surgeon
   10. Gynecologic oncologist
   11. Gynecologist
   12. Primary care provider
   13. Researcher
   14. Nurse
   15. Medical Geneticist
   16. Other (fill in)
3. Location of primary practice

[Drop-down menu of all countries]

[If USA chosen, drop down menu for each state]

1. Approximate average number of patients seen *per month* by you for initial (i.e. new patient) hereditary gastrointestinal (GI) cancer risk assessment. *Note: this question is asking your individual volume and not the practice/clinic volume.*
   1. 0-*I do not see patients for hereditary GI cancer risk assessment* [If this is chosen, have the survey skip question 5]
   2. 1-5
   3. 6-10
   4. 11-20
   5. 21-30
   6. 31-40
   7. 41-50
   8. 51-60
   9. 60+
2. What year was your practice/group established for hereditary GI cancer risk assessment?

[Drop down menu with years from 1980-present]

*We originally asked you what your typical next test would be for certain case examples without any barriers. Now, we want to know IF there are barriers and WHAT those barriers are and how this might change your response. Please answer the follow questions ASSUMING A TYPICAL CLINICAL SCENARIO in your practice (for example, type of insurance, current choice of laboratory, etc.).*

For the following case examples, screening was performed by IHC for MLH1, MSH2, MSH6 and PMS2 proteins. Paired tumor/germline testing includes both tumor sequencing and germline testing of at least the LS genes. It may also include somatic or germline testing of additional non-LS genes.

1. Your patient was diagnosed with colorectal cancer at age 45 and the tumor showed absent MSH2/MSH6. There is no family history of LS cancers. The typical next test ordered for your patient is:
2. Germline testing for MSH2 only
3. Germline testing for MSH2/MSH6/EPCAM
4. Germline testing for all five LS genes
5. Germline testing with a multi-gene panel that includes all five LS genes
6. Paired tumor/germline testing that includes LS
7. Other (write in)
8. N/A to my practice
9. Would your above answer have been different *without any barriers* (cost, insurance, access, etc.)?
   1. Yes
   2. No
10. If yes, WHAT are the barriers that might change the choice of test (check all that apply)
    1. Lack of insurance coverage
    2. Patient unable/unwilling to self-pay if insurance doesn’t cover
    3. Not enough clinical laboratories offering the test you need
    4. I don’t feel comfortable counseling patients about test results that include somatic tumor testing
    5. I don’t have flexibility to choose the next test (i.e. it is automatic at my institution or my institution has a specific policy)
    6. There is no tumor available for paired testing
    7. I do not have the time to coordinate the shipment of a tumor specimen
    8. Turn-around time for paired analysis is too long
    9. Turn-around time for multi-gene panel is too long
    10. Patient declines my initial testing recommendation
    11. Other. Please explain:
11. Your patient was diagnosed with colorectal cancer at age 45 and the tumor showed absent MSH2/MSH6. Your patient’s mother was diagnosed with colorectal cancer at 50 with no additional family history. The typical next test ordered for your patient is:
12. Germline testing for MSH2 only
13. Germline testing for MSH2/MSH6/EPCAM
14. Germline testing for all five LS genes
15. Germline testing with a multi-gene panel that includes all five LS genes
16. Paired tumor/germline testing that includes LS
17. Other (write in)
18. N/A to my practice
19. Would your above answer have been different *without any barriers* (cost, insurance, access, etc.)?
    1. Yes
    2. No
20. If yes, WHAT are the barriers that might change the choice of test (check all that apply)
    1. Lack of insurance coverage
    2. Patient unable/unwilling to self-pay if insurance doesn’t cover
    3. Not enough clinical laboratories offering the test you need
    4. I don’t feel comfortable counseling patients about test results that include somatic tumor testing
    5. I don’t have flexibility to choose the next test (i.e. it is automatic at my institution or my institution has a specific policy)
    6. There is no tumor available for paired testing
    7. I do not have the time to coordinate the shipment of a tumor specimen
    8. Turn-around time for paired analysis is too long
    9. Turn-around time for multi-gene panel is too long
    10. Patient declines my initial testing recommendation
    11. Other. Please explain:
21. Your patient was diagnosed with colorectal cancer at age 75 and the tumor showed absent MSH2/MSH6. There is no family history of Lynch cancers. The typical next test ordered for your patient is:
22. Germline testing for MSH2 only
23. Germline testing for MSH2/MSH6/EPCAM
24. Germline testing for all five LS genes
25. Germline testing with a multi-gene panel that includes all five LS genes
26. Paired tumor/germline testing
27. Other (write in)
28. N/A to my practice
29. Would your above answer have been different *without any barriers* (cost, insurance, access, etc.)?
    1. Yes
    2. No
30. If yes, WHAT are the barriers that might change the choice of test (check all that apply)
    1. Lack of insurance coverage
    2. Patient unable/unwilling to self-pay if insurance doesn’t cover
    3. Not enough clinical laboratories offering the test you need
    4. I don’t feel comfortable counseling patients about test results that include somatic tumor testing
    5. I don’t have flexibility to choose the next test (i.e. it is automatic at my institution or my institution has a specific policy)
    6. There is no tumor available for paired testing
    7. I do not have the time to coordinate the shipment of a tumor specimen
    8. Turn-around time for paired analysis is too long
    9. Turn-around time for multi-gene panel is too long
    10. Patient declines my initial testing recommendation
    11. Other. Please explain:
31. Your patient was diagnosed with colorectal cancer at age 75 and the tumor showed absent MSH2/MSH6. The patient’s mother had colorectal cancer at age 50. The typical next test ordered for your patient is:
32. Germline testing for MSH2 only
33. Germline testing for MSH2/MSH6/EPCAM
34. Germline testing for all five LS genes
35. Germline testing with a multi-gene panel that includes all five LS genes
36. Paired tumor/germline testing
37. Other (write in)
38. N/A to my practice
39. Would your above answer have been different *without any barriers* (cost, insurance, access, etc.)?
    1. Yes
    2. No
40. If yes, WHAT are the barriers that might change the choice of test (check all that apply)
    1. Lack of insurance coverage
    2. Patient unable/unwilling to self-pay if insurance doesn’t cover
    3. Not enough clinical laboratories offering the test you need
    4. I don’t feel comfortable counseling patients about test results that include somatic tumor testing
    5. I don’t have flexibility to choose the next test (i.e. it is automatic at my institution or my institution has a specific policy)
    6. There is no tumor available for paired testing
    7. I do not have the time to coordinate the shipment of a tumor specimen
    8. Turn-around time for paired analysis is too long
    9. Turn-around time for multi-gene panel is too long
    10. Patient declines my initial testing recommendation
    11. Other. Please explain:
